# Cardiac Magnetic Resonance for Ventricular Tachycardia Ablation and Risk Stratification

**DOI:** 10.3389/fcvm.2021.797864

**Published:** 2022-01-12

**Authors:** Ivo Roca-Luque, Lluis Mont-Girbau

**Affiliations:** ^1^Arrhythmia Section, Cardiology Department, Cardiovascular Clinical Institute, Hospital Clínic, Universitat de Barcelona, Barcelona, Spain; ^2^Institut d'Investigacions Biomèdiques August Pi i Sunyer (IDIBAPS), Barcelona, Spain; ^3^Centro de Investigación Médica en Red, Enfermedades Cardiovasculares (CIBERCV), Madrid, Spain

**Keywords:** ventricular tachycardia, ablation, cardiac magnetic resonance, SCAR, electroanatomical mapping

## Abstract

Ventricular tachycardia is the most frequent cause of sudden cardiovascular death in patients with structural heart disease. Radiofrequency ablation is the treatment cornerstone in this population. Main mechanism for structural heart disease-related ventricular tachycardia is re-entry due to presence of slow conduction area within the scar tissue. Electroanatomical mapping with high density catheters can elucidate the presence of both scar (voltage maps) and slow conduction (activation maps). Despite the technological improvements recurrence rate after ventricular tachycardia ablation is high. Cardiac magnetic resonance has demonstrated to be useful to define the location of the scar tissue in endocardium, midmyocardium and/or epicardial region. Furthermore, recent studies have shown that cardiac magnetic resonance can analyse in detail the ventricular tachycardia substrate in terms of core scar and border zone tissue. This detailed tissue analysis has been proved to have good correlation with slow conduction areas and ventricular tachycardia isthmuses in electroanatomical maps. This review will provide a summary of the current role of cardiac magnetic resonance in different scenarios related with ventricular tachycardia in patients with structural heart disease, its limitations and the future perspectives.

## Introduction

Ventricular tachycardia (VT) is the most common cause of sudden cardiovascular death (SCD) (1). Catheter ablation has become a standard treatment for VT in patients with structural heart disease (2, 3). Activation and entrainment mapping can be performed in only 30–40% of cases due to hemodynamically non-tolerated VTs. Substrate-based ablation, based on the identification of arrhythmic substrates through maps in sinus or paced rhythm, have been developed in the past few years, initially to treat poorly tolerated infarct-related VTs (4) but currently, it has been established as the cornerstone for VT ablation in patients with structural heart disease, with better results in terms of VT recurrence rate than strategies based only on activation mapping (5, 6). The main mechanism behind structural heart disease-related VT is the presence of a re-entrant circuit (7–9). This circuit is formed by the presence of a slow conduction area (border zone tissue, BZ) within the non-excitable scar (core scar) that connects to the healthy non-scarred myocardium, leading to re-entry. These regions are also called conducting channels (CCs). These CCs can be accurately identified using electroanatomical maps (EAMs) obtained during mapping and ablation (9–12). In these areas, reduced conduction velocity and non-uniform anisotropic conduction give rise to areas of delayed, fractionated electrical activity that can persist even after the inscription of the QRS complex during sinus or paced rhythm. Several studies have demonstrated the relationship between these electrograms, the so-called late potentials, or local abnormal ventricular activity and VT. Indeed, many authors have suggested that complete elimination of the VT substrate results not only in tachycardia non-inducibility but also in less VT recurrences in the long-term follow-up (13–16).

Despite the better knowledge of VT substrates and technological improvements in mapping systems and ablation strategies, the VT recurrence rate in structural heart disease-related VT is still high (14, 17–19). Some of the recurrences can be related to new circuits due to the progressive nature of the underlying heart disease. However, studies on redo procedures have shown that in more than half of the cases VT is related to the same susbtrate as in the index procedure (20). There is a clinical need to improve the efficacy of catheter ablation for VT. In this context, cardiac magnetic resonance imaging (CMR) may play a role in the optimisation of VT ablation.

Better knowledge of VT substrates is still necessary to improve ablation outcomes. In this sense, advanced imaging techniques such as CMR with late gadolinium enhancement (LGE-CMR) have been able to identify scar tissue both in animal models and in humans (21–23) and they can identify not only the total scar but also the heterogeneous tissue (BZ) that, as stated before, can be the substrate for slow conduction and re-entry. This review aims to review the role of LGE-CMR in VT ablation.

## Fundamentals of LGE-CMR in Detecting VT Substrate

LGE is a result of regional differences in myocardial extracellular volume, differential uptake, and washout patterns within the extracellular space, and it is seen in non-healthy myocardium as the washout time in these areas is longer than those in healthy tissue.

**Figure 1:**
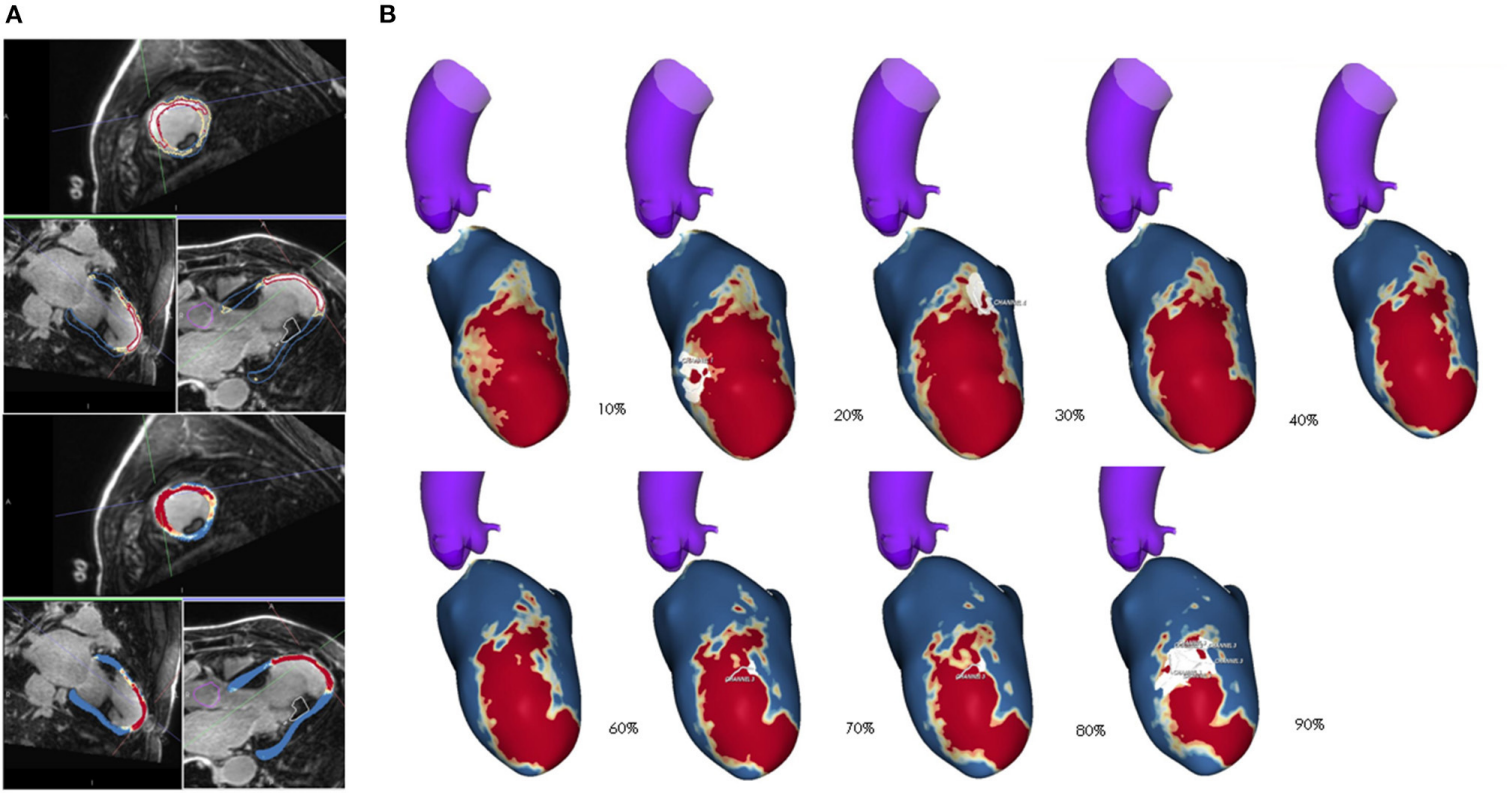
**(A)** Raw images of cardiac magnetic resonance (CMR) in a patient with chronic anterior myocardial infarction. The bright area seen is due to late gadolinium enhancement (LGE). Automatic segmentation of endocardium and epicardium with detection of the core scar is shown in red (intensity of LGE >60% of the area with maxim intensity signal of LGE), healthy tissue is shown in blue (intensity of LGE <40% of the area with maxim intensity signal of LGE) and the border zone is shown in yellow (areas with intermediate LGE intensity). **(B)** Three-dimensional reconstruction of CMR is shown from endocardium (layer 10%) to epicardium (layer 90%) with large anterior transmural myocardial infarction, with area of channels (white) of intermediate tissue (yellow) crossing the core scar (red).

The role of LGE-CMR in detecting fibrosis had been described in the late 90's (24). From the very beginning, a clear relationship between LGE and arrhythmogenicity was demonstrated, as LGE was able to identify heterogeneous scars (21–23). Furthermore, some groups started to analyse the ability of LGE-CMR to create maps of the myocardium based on the different pixel intensity signal of LGE depending on the amount of the scar of the myocardium, called pixel signal intensity maps (PSI). The first studies were performed with 1.5-T CMR scans (25) in patients with chronic myocardial infarction. They compared the LGE-CMR of patients that presented VT with those of patients with without VT. In addition, the relationship of invasive voltage maps with PSI maps of LGE-CMR was analysed. This study confirmed that channels of heterogeneous tissue inside the scar connecting to the healthy tissue identified in LGE-CMR were more common in patients with VT (88 vs. 33%), suggesting a relationship with arrhythmogenic risk. More importantly, it was demonstrated that these channels of LGE-CMR PSI maps corresponded to those identified by endocardial voltage mapping in the EAM. These results were confirmed by a study of our group (26), with 3-T scans and a threshold signal intensity of 60% in the PSI map was established to detect the core with a good correlation (80%) with EAMs. Figure 1 shows an example of the three-dimensional reconstruction of the PSI LGE-CMR map. Furthermore, subsequent studies with the 3-T (27) scans and a dedicated imaging post-processing software confirmed the utility of LGE-CMR in detecting CCs not only compared with electroanatomical voltage maps but also by analysing the electrogram characteristics in the CCs. The CCs detected by PSI maps were the areas of late potentials in the EAMs; therefore, slow conduction was confirmed. In addition, with the ability of the LGE-CMR to differentiate the scar tissue in the endocardium, midmyocardium, and epicardium, it was shown that the scar has a three-dimensional structure and LGE-CMR can detect the three-dimensional pathway of the CCs with a higher concordance and better detection of the BZ than the conventional two-dimensional LGE-CMR.

## Clinical Applications of LGE-CMR in VT Ablation

### Pre-procedural CMR

Pre-procedural LGE-CMR is gaining widespread applicability as an assistance tool in ablation procedures because it can facilitate procedural planning, scar mapping, and ablation (28–30), as well as the evaluation of the risk of recurrences after ablation (31).

However, the majority of patients scheduled for VT ablation have an implantable cardioverter defibrillator (ICD). This issue represents a major limitation of affecting image quality with conventional LGE-CMR. The use of wideband sequences can overcome this limitation by minimising device-related artefacts, making LGE-CMR imaging robust for myocardial characterisation under these conditions (32–34). A good correlation between wideband LGE-CMR and EAM has recently been demonstrated in previous work by our group (35).

**Figure 2:**
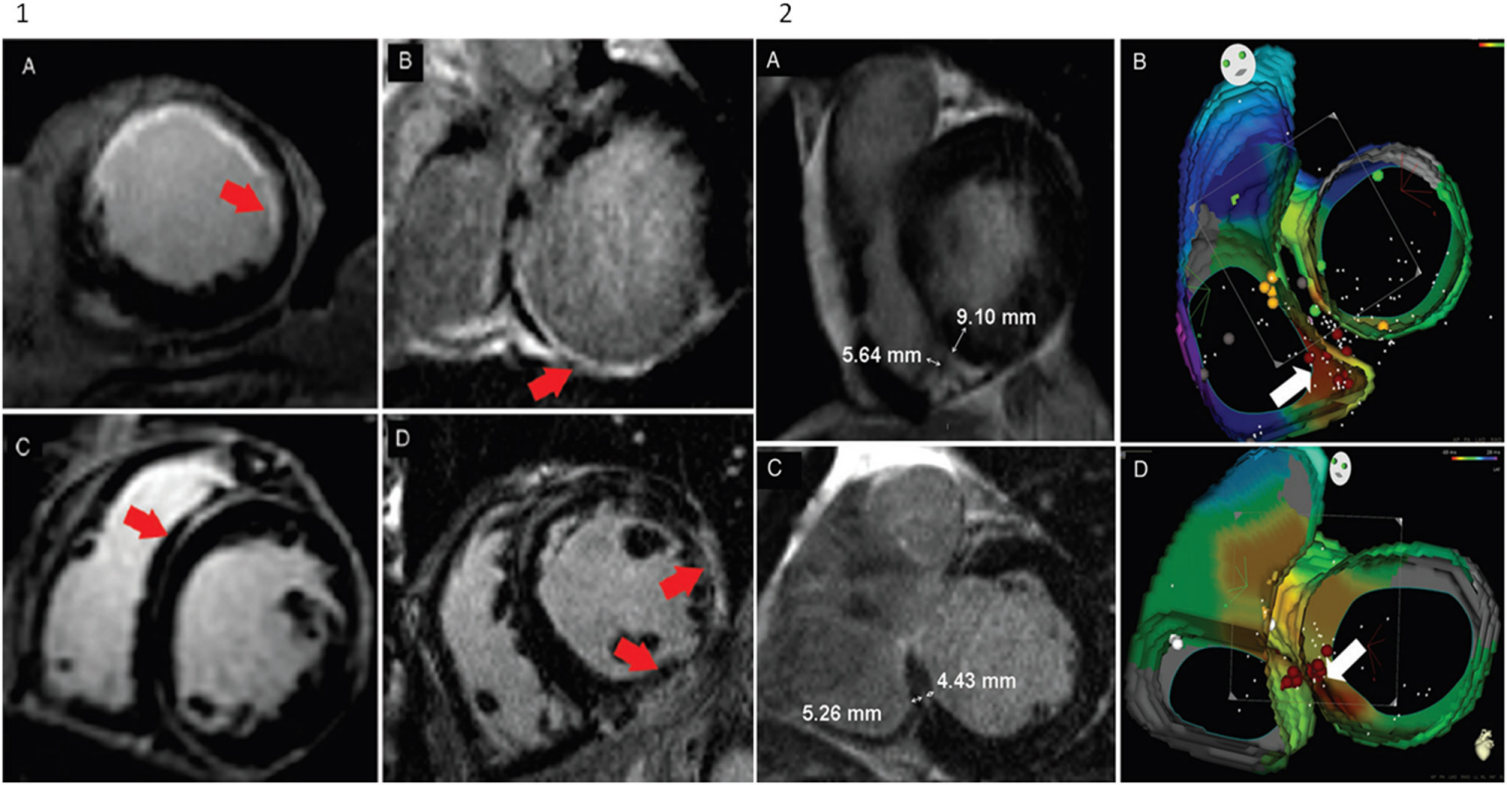
Adapted from Andreu et al. (29). Panel 1: Pattern distribution of hyperenhancement in cardiac magnetic resonance images. **(A)** Endocardial hyperenhancement in left ventricle the anterior wall. **(B)** Transmural hyperenhancement in the left ventricle posterior wall and endocardial hyperenhancement of the posteroseptal wall. **(C)** Mid-myocardial hyperenhancement in the anteroseptal wall. **(D)** Epicardial hyperenhancement in the left ventricle lateral wall. Panel 2: Cardiac magnetic resonance and electroanatomical map of two patients with mid-myocardial hyperenhancement at the successful ablation site. **(A,B)** Endocardial ablation of premature ventricular contractions originating from the right ventricle. **(A)** The distance to the boundary of the hyperenhancement region was shorter in the right ventricle than in the left ventricle. **(B)** Radiofrequency ablation of the right ventricle was performed. **(C,D)** Endocardial ablation of the left ventricle. **(C)** In this case, the distance to the boundary of the hyperenhancement region was shorter from the left ventricle than from the right ventricle. **(D)** A previous unsuccessful radiofrequency ablation was attempted in the right ventricle. After mapping, the left ventricle the maximum precocity of the left ventricle septum was obtained, and radiofrequency ablation eliminated premature ventricular contraction.

The use of LGE-CMR to plan the ablation procedure is especially useful to assess the scar location and to determine the best ablation approach. In this sense, a non-randomised study by Acosta et al. (36) proved that in ischemic patients with epicardial substrate, identified by imaging techniques, who underwent epicardial access had better results in terms of VT recurrence than patients with epicardial substrate who underwent the endocardial approach only. The location of the scar in non-ischemic cardiomyopathy (NICM) patients has also been demonstrated to be useful for the ablation procedure (28), since the location is related to the type of tachycardia and the ablation approach. In this sense, the usefulness of CMR in deciding the ablation approach was demonstrated in a larger study involving 80 patients with both ischemic cardiomyopathy (ICM)and NICM. In that study, the endocardial or epicardial approach was based on imaging, and the access to intramural substrate (left and/or right ventricle) was also decided depending on the distance of the scar to the left and right endocardium (Figure 2) (29).

**Figure 3:**
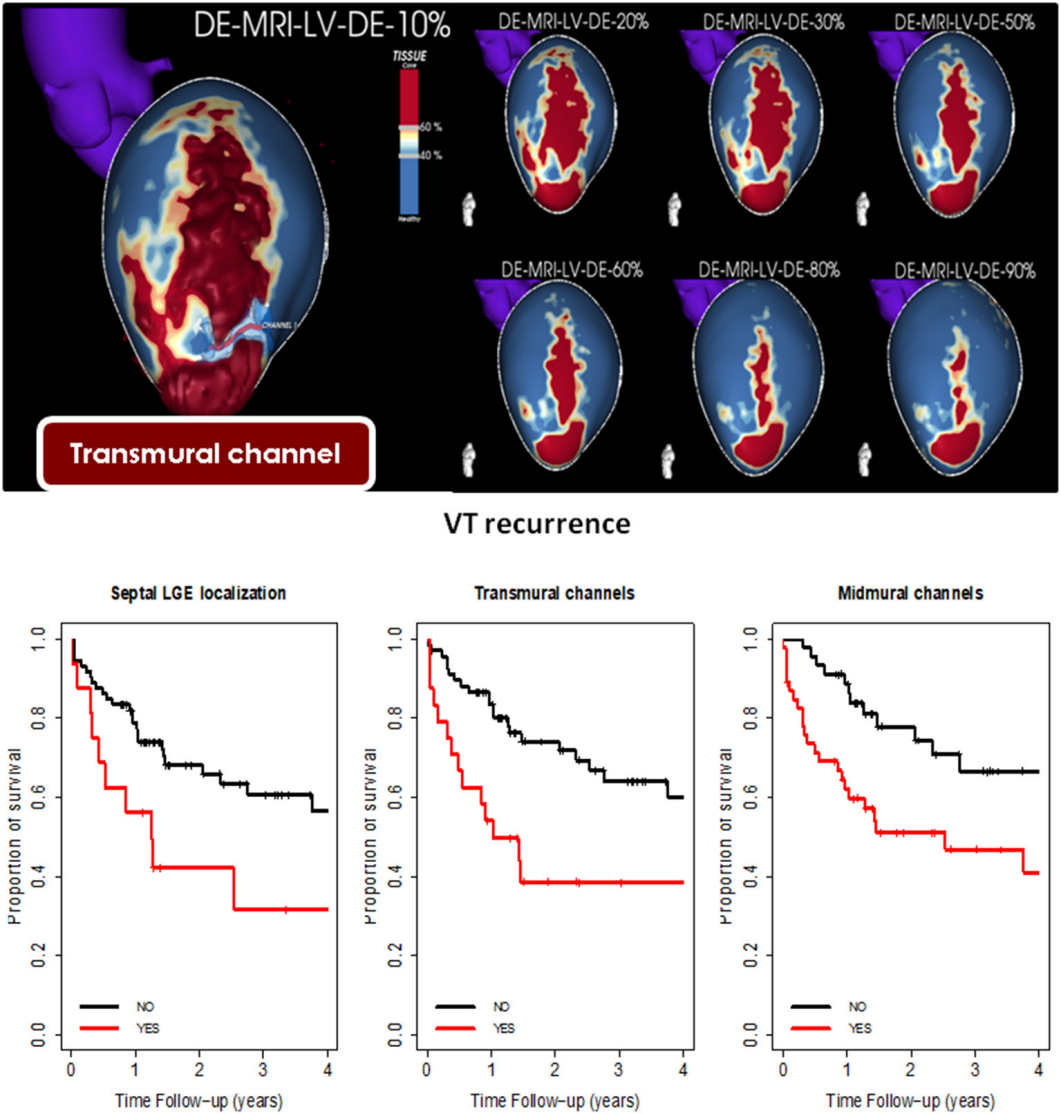
Adapted from Quinto et al. (31). Upper panel: Example of transmural channel in pixel signal intensity (PSI) map of three-dimensional late gadolinium enhancement-cardiac magnetic resonance (LGE-CMR) reconstruction of the left ventricle. The core scar is shown in red, border zone in yellow, and healthy tissue in blue. A clear transmural large anteroapical scar with a channel of border zone in the apical-lateral is seen from the endocardial layers (20–30%) to the epicardial (80–90%). The bottom panel shows the Kaplan-Meier curve for ventricular tachycardia-free survival according to the presence of septal substrate, transmural channels, and midmural channels.

Pre-procedural CMR has already been proven to be related to recurrence rate. Quinto et al. (31) analysed 110 consecutive patients who underwent VT ablation with pre-procedural LGE-CMR, and several factors identified by CMR were clearly related with a higher recurrence rate: BZ and total scar mass, transmural and midmural channels, and septal substrate (Figure 3). In a previous smaller study (37), scar area was also related with VT recurrence, and a more recent study (38) including patients with NICM (25 patients) also confirmed that septal LGE extension was related to a higher VT recurrence rate.

### Intraprocedural CMR

After the first studies confirming the ability of CMR to detect myocardial scars and those showing the usefulness to plan the ablation approach, some studies started to assess the feasibility of integration of CMR into the navigation system.

**Figure 4:**
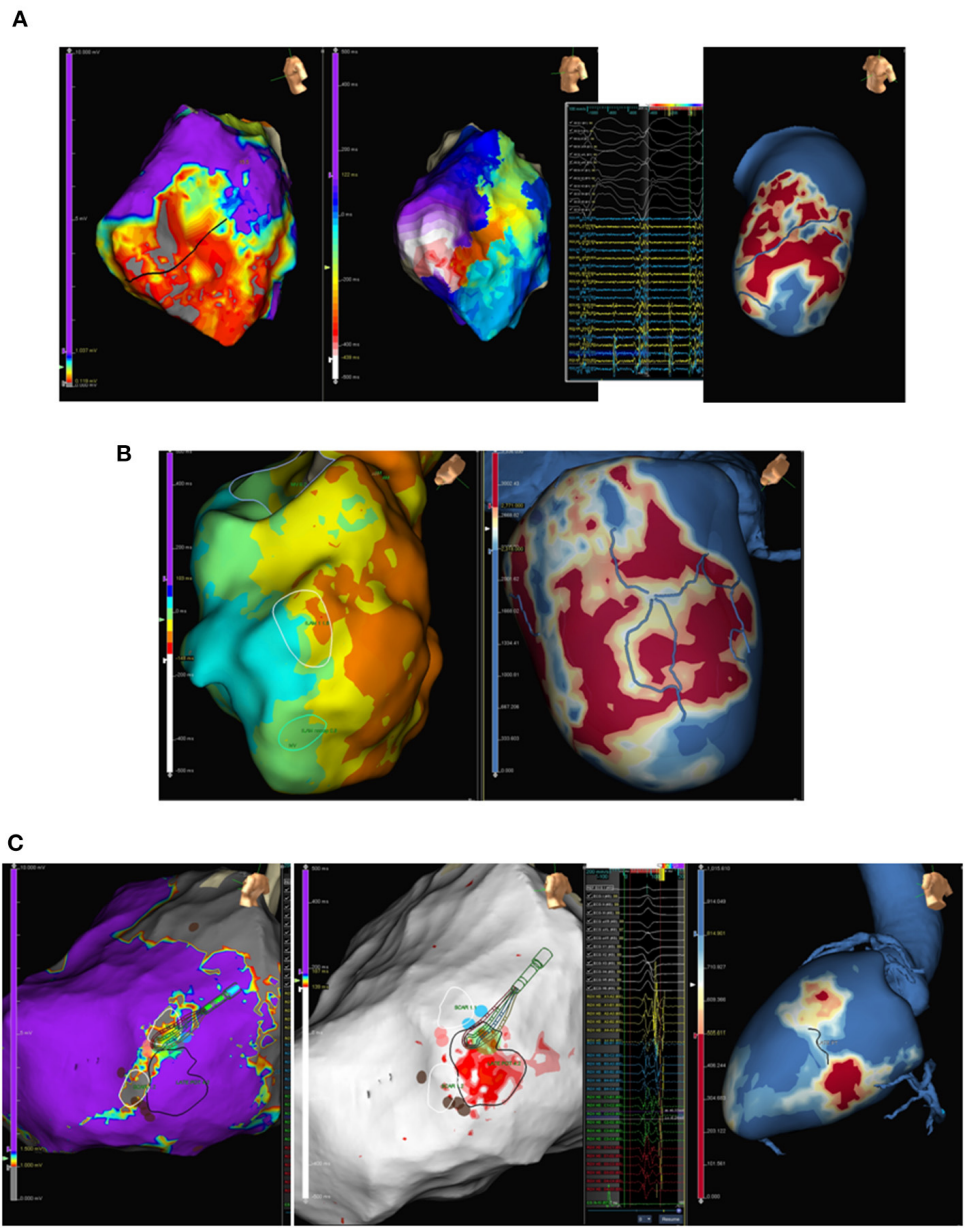
Examples of correlation of three-dimensional pixel signal intensity (PSI) maps reconstruction from late gadolinium enhancement-cardiac magnetic resonance (LGE-CMR) (red: core scar, yellow: border zone, blue: healthy tissue: blue) with different electroanatomical (EAM) maps. **(A)** From left to right: EAM high density voltage map showing anterolateral scar (core scar: grey, border zone: red, healthy tissue: purple), ventricular tachycardia with a figure-of-eight circuit using border zone inside the scar (black line in the voltage map) with diastolic electrograms during ventricular tachycardia (VT); and PSI LGE-CMR map with clear conducting channel (blue line) at the same region as compared to the EAM voltage and activation map. **(B)** Left panel shows sinus rhythm isochronal activation map with an area of isochronal crowding (white circle) suggesting an area of deceleration zone. Right panel shows LGE-PSI map with an area of a very ramified channel (blue lines) inside a scar in the same area of deceleration zone in sinus rhythm EAM map. **(C)** Epicardial high density voltage map (left panel) and late potentials map (centre panel) with two small scars (right panel, grey area), intermediate tissue between scars, and area (red) of late potentials corresponding to border zone between scars. Right panel shows epicardial LGE-CMR PSI map (layer 80%) with the same two scars detected in the voltage map.

The first studies comparing scar extension in EAM with CMR PSI maps were performed using side-by-side comparisons (39) or using post-processing software (28, 40). Most of those studies included mainly ischemic patients and analysed the correlation between scar area and CCs in CMR and EAM with an average correlation of 80%. Indeed, some of the studies (28, 40, 41) also showed that critical VT isthmuses were located in the transition of the BZ/core area and/or close to the transmural scar located in the CMR. After a good correlation of CMR and EAM was stated, integration of CMR images into the navigation system during the ablation procedure (not only for research purposes with post-processing software) was proved to be feasible (28, 42, 43). These studies not only confirmed the good correlation between scars detected in CMR and voltage EAM maps but also showed that scars in CMR were larger than those in EAM. More importantly, some of the VT isthmuses were located in these areas of the scar in CMR but with normal voltage in EAM (40). These areas of scarring not detected in EAM were also frequent areas with hidden slow conduction identified with the multiple extrastimuli technique (44), suggesting a better arrhythmogenic substrate characterisation of the scar by the CMR. Similarly, the capability of LGE-CMR to detect arrhythmogenic substrates seems to be superior to EAM in NICM if only voltage maps are used in EAM. Glashan et al. (45) did not find a good correlation between fibrosis and a single voltage cut-off (unipolar or bipolar) in a study that compared EAM with histological findings. This could be explained by the higher proportion of non-endocardial substrates in NICM and by the different patterns of fibrosis in this population. Figure 4 shows different examples of correspondence of PSI LGE-CMR three-dimensional maps with different EAM maps (voltage maps, isochronal late activation time maps and VT activation maps).

**Figure 5:**
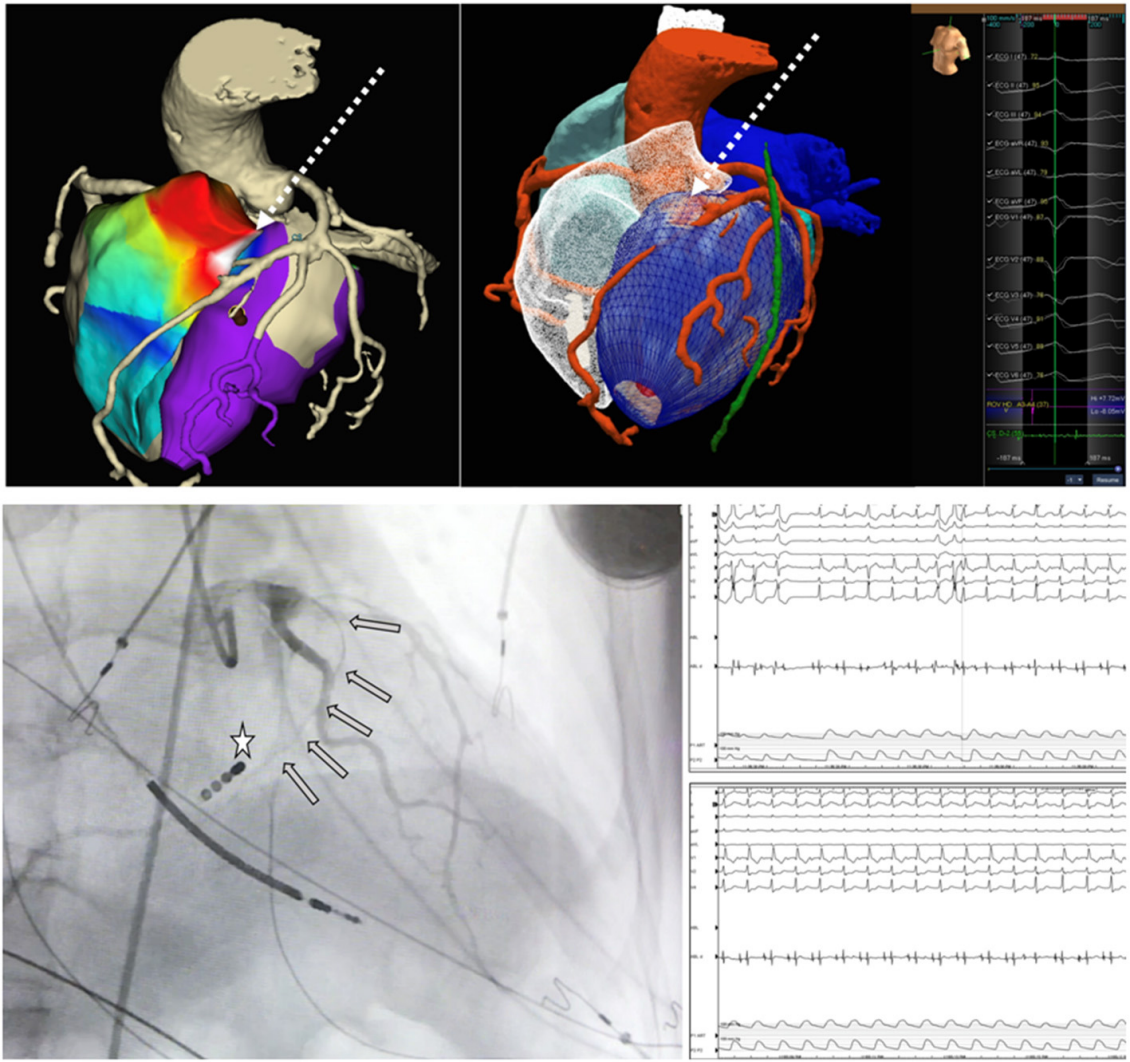
Adapted from Roca-Luque et al. (30). Pace-map electroanatomical map (EAM) of right ventricle (RV) (anterior view) with the highest concordance with ventricular tachycardia morphology (92%) in the anterior RV septum. Ablation as unsuccessful from both right and left ventricle. EAM is merged with pre-procedural cardiac tomography (CT) scan and a septal artery is observed irrigating the area of best pace-map. Centre panel shows only CT scan with septal artery irrigating an area of intramural eptal scar (red) corresponding to the area of best pace-map. Bottom panels show septal alcolholisation of the culprit area according pre-procedural imaging The wire inside septal artery (grey arrows) is shown in front of catheter tip (white star) in the RV septum. After alcholisation at that region (bottom right panel) ventricular tachycardia stops.

Unfortunately, very few studies have analysed the influence of imaging on ablation success. Small observational studies (30) showed the usefulness of image integration for complex patients in whom special techniques, such as septal alcholisation, differ from conventional radiofrequency ablation (Figure 5). After these studies in very specific populations, some groups started to analyse the potential role of CMR in improving ablation success in routine VT ablation procedures in both ICM and NICM patients. Despite several studies proving the feasibility of the integration of CMR images into a navigation system to assist VT ablation, few studies comparing image-guided VT ablation with standard procedures have been published. Overall, only five studies (non-randomised) (46–50) and one meta-analysis (51) have been published. Some of the studies (48, 49) suggest that the use of imaging is associated with shorter procedure time, shorter radiofrequency time, and shorter fluoroscopy time. More importantly, all the studies and the meta-analysis showed a benefit in VT recurrence in the imaging-aided ablation group. However, randomised trials have not been conducted in this field.

### CMR as a Risk Stratification Tool in Patients at Risk of Ventricular Arrhythmias

Currently, clinical practise guidelines for recommending an ICD for the primary prevention of SCD are based only on the left ventricular ejection fraction (LVEF). ICD is indicated in patients with LVEF < 35%. Although LVEF can identify a subgroup of patients at risk of SCD, appropriate ICD therapy is documented in only one third of the patients (52), so its use as the sole criterion for implanting an ICD implies overtreatment of a high number of patients. Thus, tools for improving the prediction of arrhythmic risk are needed.

**Figure 6:**
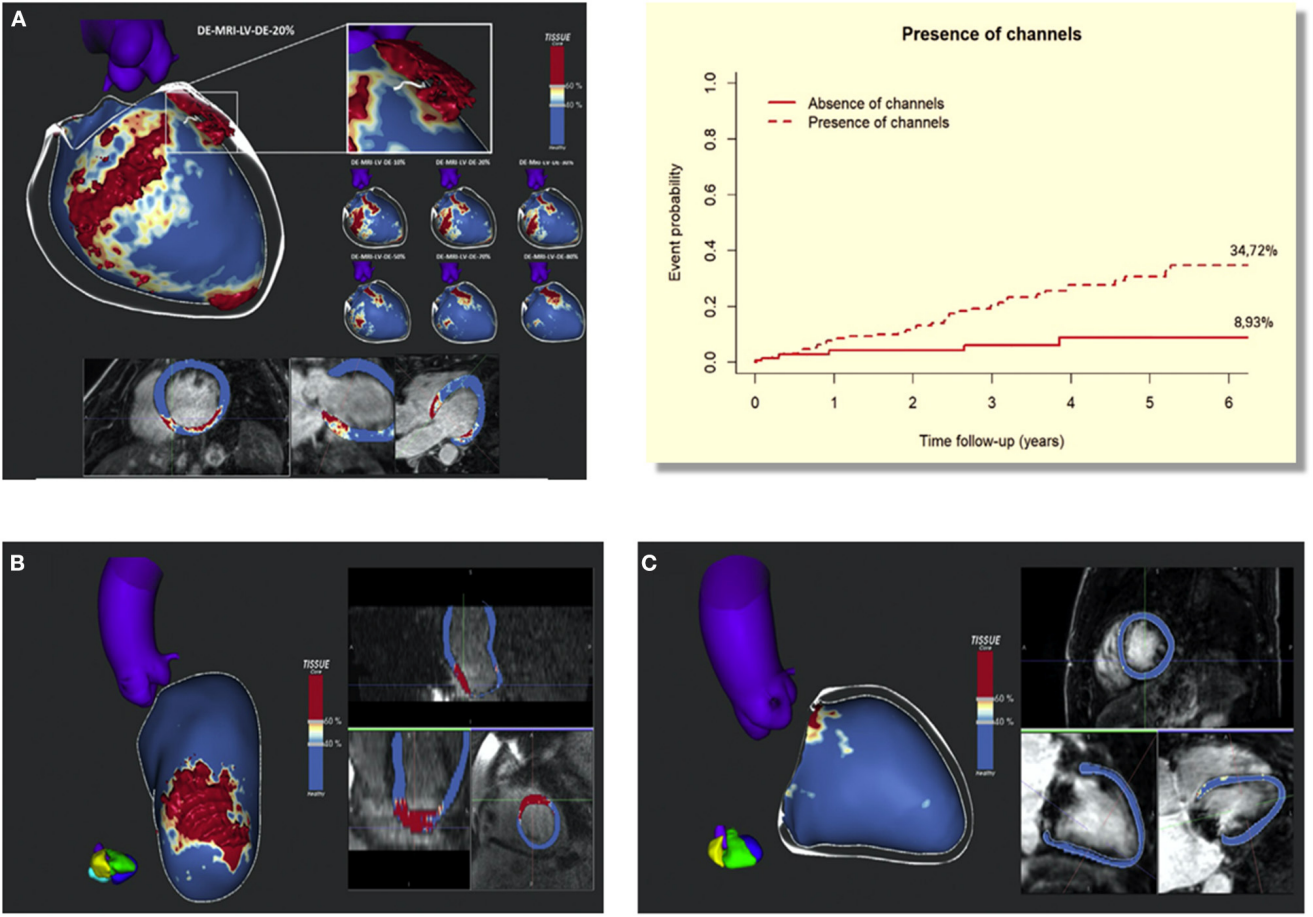
Adapted from Sánchez-Somonte et al. (54). **(A)** Late gadolinium enhancement-cardiac magnetic resonance (LGE-CMR) reconstruction of the left ventricle (LV) with a posteroseptal scar (core in red, BZ in white, and healthy myocardium in blue). A white line is drawn over the surface, representing a conducting channel. We can see the substrate evolution through different layers, from the endocardium (10–30%) to the epicardium (70–90%), with a defined channel in different layers. **(B)** LGE-CMR reconstruction of the LV with an anterior scar. In this case the scar is very homogeneous (mainly composed of core tissue) compared with the scar in panel **(A)** and it has no conducting channels. **(C)** LGE-CMR reconstruction of the LV without scar. BZ, 5 border zone; LGE-CMR, 5 late gadolinium enhanced cardiac magnetic resonance; LV, 5 left ventricle. Upper right panel shows cumulative incidence functions for appropriate therapies depending on scar mass >10 g and the present channels. At 6-year follow-up, 37.65% of patients with channels and scar mass > 10 g reached the primary end point compared with 2.78% of patients without channels and scar mass < 10 g.

Several studies have demonstrated the usefulness of LGE-CMR in this field in patients with ICM. Infarct size assessed by LGE-CMR is an independent predictor of arrhythmic events (53). However, in a recent study by our group (54) we prospectively analysed 200 patients (mainly ischemic patients) with primary prevention ICD indication (LVEF <35%), and despite 83% of them having undergone LGE-CMR, only 20% of them had ICD-appropriate therapies in long-term follow-up. In this sense, a more detailed analysis of LGE-CMR images assessing the amount of scar, BZ, and the presence of channels can improve risk stratification. In our study, a scar mass > 10 g (hazard ratio: 4.74, 95% CI: 1.12–20; *p* = 0.034) and the presence of CC (hazard ratio: 4.07, 95% CI: 1.59–10.4, *p* = 0.003) were strongly associated with appropriate ICD therapies and, in the same line, only one patient without CCs and a scar mass < 10 g had appropriate ICD therapy, representing that the rate of ICD therapies in this group was <3% in 5 years (Figure 6).

The usefulness of CMR in assessing arrhythmic risk has already been proven in patients with NICM. In the DANISH study's substudy, the DANISH-MRI study (55) fibrosis was shown to be an independent predictor for all-cause mortality and arrhythmic events. Similarly, an observational study conducted by Gutman et al. (56) demonstrated a survival benefit associated with the implantation of an ICD for primary prevention in NICM only for patients with scar tissue on CMR, Finally, in a retrospective, very large observational study involving 1,165 patients with dilated cardiomyopathy who underwent LGE-CMR (57), LGE in CMR was found to be a very strong predictor of ventricular arrhythmias and sudden death. In addition, the presence of LGE but also specific LGE patterns (epicardial, transmural, or combined septal and free wall LGE) were found to be predictive of arrhythmic events and were considered high-risk LGE patterns. These results confirm, as in our previously mentioned study in ICM patients, that a more detailed analysis of LGE-CMR has added value for risk stratification in the presence of LGE.

## Limitations and Future Perspectives

The main limitation of LGE-CMR in most centres is the low spatial resolution. In this sense, a 3-T scan can have a maximum voxel resolution of 1.4 × 1.4 × 1.4 mm. CCs are three-dimensional structures, and hypothetical structures can be composed of very small bundles of surviving myocytes. It has been described that re-entry can be sustained even in a corridor of <200 micrometres (7). Therefore, these very small channels could be detected with the current LGE-CMR spatial resolution. Nevertheless, technical improvements in this field are expected in the near future.

Image integration into the navigation systems is still an unsolved issue. First of all, there is no homogenous method to merge the CMR images into the navigation system. While some groups perform an EAM of right ventricle outflow tract and pulmonary artery to be used as a landmark for merging, other groups perform a map of aortic root and cannulate the left main with ablation catheter for merging purposes. A more homogenous and simple method would be useful to expand this technique to more electrophysiology labs. In addition, the registration error reported within all the studies have generally been in the range of 3–5 mm. As we stated before, when addressing the limitation of spatial resolution of CMR, this registration error could be an issue in performing a real exclusive CMR-guided VT ablation. Due to all of these issues and the fact that, imaging does not contain electrical information, there is a global consensus that LGE-CMR should be considered an excellent complementary tool for helping VT ablation, but it cannot substitute electrophysiological information provided by mapping catheters at present.

Another problem regarding image integration is the elapsed time between LGE-CMR acquisition and VT ablation. As myocardiopathy is a progressive disease, the arrhythmogenic substrate can be different from the time when LGE-CMR is performed to the ablation date. To overcome this issue, real-time CMR-guided ablation procedures have been described (58–60) but the main case series have been described in atrial procedures. Diagnostic studies have been performed in animal models to characterise intracardiac electrograms within ventricular scars under real-time magnetic resonance-guidance (61). Oudeneye et al. (61) first described the use of active catheter tracking to navigate catheters into the left ventricle in swine, to record intracardiac electrograms and to acquire three-dimensional voltage maps. Although conceptually, real-CMR has potential benefits in VT ablation, it requires substantially changing the workflow and getting used to altered signals and electrocardiography in the CMR environment; and, to our knowledge, no study has been published in humans in the field of VT ablation.

Regarding the evaluation of benefits of LGE-CMR in both VT ablation and risk stratification, all of them have been described in this manuscript. However, in both fields, all of the evidence is based on observational studies. Therefore, future randomised trials are needed to confirm the usefulness of LGE-CMR in both fields.

Finally, LGE-CMR has a potential role in the evaluation of ablation lesions. The use of LGE-CMR to evaluate radiofrequency ablation lesions has been studied in the acute phase in both animal models (62) and humans (63). Ablation lesions are detected in the LGE-CMR as an area of no enhancement due to microvascular obstruction, also called the dark core in post-contrast T1-weighted imaging. In a recent paper, these dark core areas were related to the area of the ablation lesion (64). However, these data are based on very small series and are still controversial, as animal studies suggest that visualisation of the dark core depends on many factors such as the presence of a previous scar, the elapsed time between the ablation procedure, and the LGE-CMR acquisition, as well as on the delay from contrast LGE injection to image acquisition. Large prospective studies are needed to confirm the role of LGE-CMR in the analysis of ablation lesions and its relationship with clinical endpoints.

## Author Contributions

IR-L and LM-G contributed to the initial design of the paper, made a critical revision of the article, and approved the final version to be published. IR-L written the manuscript. All authors contributed to the article and approved the submitted version.

## Funding

This work was supported in part for a grant n° PI20/00693 of Instituto de Salud Carlos III.

## Conflict of Interest

LM-G reports fees as consultant, lectures and advisory board for Abbott Medical, Boston Scientific, Medtronic, Biosense Websters and he was shareholder of Galgo Medical, S. L. IR-L reports fees as consultant for Abbot Medical and Boston Scientific.

## Publisher's Note

All claims expressed in this article are solely those of the authors and do not necessarily represent those of their affiliated organizations, or those of the publisher, the editors and the reviewers. Any product that may be evaluated in this article, or claim that may be made by its manufacturer, is not guaranteed or endorsed by the publisher.

## Abbreviations

BZ: Border zone tissues
CC: Conducting channel
CMR: Cardiac magnetic resonance
EAM: Electroanatomical map
ICD: Implantable cardioverter defibrillator
ICM: Ischemic cardiomyopathy
LGE: Late gadolinium enhancement
LVEF: Left ventricular ejection fraction
NICM: Non-ischemic cardiomyopathy
PSI: Pixel signal intensity
SCD: Sudden cardiovascular death
VT: Ventricular tachycardia.
